# Serological responses to SARS-CoV-2 in urban and rural Ghana: Antibody waning and implications for long-term population immunity

**DOI:** 10.1371/journal.pone.0348281

**Published:** 2026-05-28

**Authors:** George Agyei, Philip El-Duah, Rexford Mawunyo Dumevi, Therese Muzeniek, Sherihane Aryeetey, Brice Armel Nembot Fogang, Jonathan M. Gmanyami, Augustina Angelina Sylverken, Yaw Adu-Sarkodie, Richard Odame Phillips, Michael Owusu, Christian Drosten

**Affiliations:** 1 Department of Clinical Microbiology, Kwame Nkrumah University of Science and Technology, Kumasi, Ghana; 2 German West African Centre for Global Health and Pandemic Prevention, Kumasi, Ghana; 3 Kumasi Centre for Collaborative Research in Tropical Medicine, Kwame Nkrumah University of Science and Technology, Kumasi, Ghana; 4 Institute of Virology, Charité-Universitätsmedizin Berlin, Berlin, Germany; 5 Department of Theoretical and Applied Biology, Kwame Nkrumah University of Science and Technology, Kumasi, Ghana; 6 School of Medicine and Dentistry, College of Health Sciences, Kwame Nkrumah University of Science and Technology, Kumasi, Ghana; 7 Department of Medical Diagnostics, Kwame Nkrumah University of Science and Technology, Kumasi, Ghana; 8 German Centre for Infection Research, Berlin, Germany; University of the Witwatersrand, SOUTH AFRICA

## Abstract

**Background:**

Ghana exhibits geographic variation across northern and southern regions alongside differences between urban and rural communities, which may influence SARS-CoV-2 exposure and immune response. This study aimed to assess the differences in seroprevalence, and seroconversion status after exposure to SARS-CoV-2 in selected urban and rural Ghanaian communities.

**Methods:**

A longitudinal study design was employed. Serum samples (n = 987) were collected during a baseline survey (August 2023–February 2024) with longitudinal follow-up (n = 212) sampling after one year (August 2024-February 2025) among consenting community participants aged ≥10 years selected through household-based sampling in urban and rural settings. Socio-demographic data, clinical symptoms, and vaccination status were collected using structured questionnaires. Serum samples were inactivated and tested with semi-quantitative Anti-SARS-CoV-2 IgG ELISA assays targeting spike (S) and nucleocapsid (N) proteins. Presence of SARS-CoV-2 neutralizing antibodies in serum was determined by a surrogate virus neutralization assay.

**Results:**

Of 987 participants aged 10–88 years (67.3% female), SARS-CoV-2 seroprevalence was comparable between urban (47.4%) and rural (47.7%) areas. Among the 212 participants followed longitudinally, 50.9% (108 participants) had both infection or vaccine-induced anti-spike and infection-induced anti-nucleocapsid antibodies after one year, indicating sustained immune responses from prior exposure or vaccination. There was a significant increase in paired spike and nucleocapsid antibody responses between baseline and follow-up (McNemar’s test, χ²(1) = 104.00, p < 0.0001), reflecting ongoing immune boosting despite evidence of antibody waning. Neutralizing antibodies, were detected in 147/148 (99.3%) individuals at timepoint-1 and in all 12 (100%) selected individuals for follow-up at timepoint-2, demonstrating robust functional immunity.

**Conclusion:**

This study demonstrates widespread serological evidence of prior SARS-CoV-2 exposure and vaccine-induced immunity in both urban and rural Ghana. Functional neutralising antibodies were detected in a longitudinal subset, suggesting persistence of antibody-mediated protection in some individuals. Larger longitudinal studies with repeated functional immune assessments are needed to better define durable protection in West African settings.

## 1. Introduction

Ghana and neighbouring West African countries have experienced heterogeneous SARS-CoV-2 transmission shaped by cross-border mobility, urbanisation, and differences in access to testing and healthcare. In Ghana, routine case-based surveillance is likely to underestimate cumulative exposure because many infections are asymptomatic or mildly symptomatic and because testing coverage has varied over time and place. As a result, population-based serological data are essential to characterise true exposure patterns and identify potential disparities between communities.

Coronavirus disease 2019 (COVID-19), caused by the novel severe acute respiratory syndrome coronavirus 2 (SARS-CoV-2), first emerged in late 2019, Wuhan, China [1]. Following its rapid global spread, the World Health Organization (WHO) declared COVID-19 a pandemic in early 2020 [2]. By May 2022, over 500 million confirmed cases and more than 6 million deaths had been recorded globally, with over 11.5 billion vaccine doses administered [3]. Common symptoms of COVID-19 ranged from fever, dry cough, and anosmia to gastrointestinal disturbances, headaches, and musculoskeletal pain [4,5]. Severe cases often present with complications such as lymphopenia, secondary bacterial infections, acute respiratory distress syndrome (ARDS), thrombosis, myocardial injury, and multi-organ dysfunction [6,7]. As a respiratory infection, COVID-19 adds to the global burden of acute respiratory tract infections (ARIs), which have long posed a significant public health challenge worldwide [8]. Like other ARIs often caused by viruses such as influenza, respiratory syncytial virus (RSV), and human coronaviruses, COVID-19 necessitates robust surveillance and response systems.

SARS-CoV-2 is a highly transmissible and pathogenic betacoronavirus, distinct from previous human coronaviruses due to its broad tissue tropism, efficient human-to-human transmission, and ability to evade immune responses [9]. The virus’s spike protein, which binds to the angiotensin-converting enzyme 2 receptor (ACE2), has undergone significant mutations, leading to the emergence of multiple variants with increased transmissibility and, in some cases, reduced sensitivity to immune protection from prior infection or vaccination [10–13]. These evolutionary changes have complicated efforts to control the pandemic and underscore the importance of monitoring both infection- and vaccine-induced immunity over time. The rapid development and deployment of vaccines have been critical, but the continued evolution of SARS-CoV-2, including the emergence of variants of concern, poses ongoing challenges for public health, especially in regions with variable vaccine coverage and healthcare access [10,12,13]. Therefore, robust serological surveillance is essential to track population immunity, guide vaccination strategies, and inform responses to future outbreaks.

Serological surveillance provides crucial insights into the transmission dynamics and immune responses associated with SARS-CoV-2 and other respiratory viruses [9]. Seroprevalence studies are vital for assessing population-level exposure, especially in identifying asymptomatic and mildly symptomatic infections often missed by PCR testing [14]. Longitudinal serological studies not only capture trends in exposure over time but also shed light on the duration and waning of antibody responses [15], which is critical for informing long-term surveillance strategies and pandemic preparedness. SARS-CoV-2 serological testing involves the quantification of anti-S, anti-NCP, and neutralising antibodies, which provide insights into prior SARS-CoV-2 exposure and support outbreak monitoring [16]. Anti-S IgG, targeting the spike protein's receptor-binding domain (RBD), indicates immunity from vaccination or infection, crucial for preventing severe disease. Anti-NCP IgG confirms past natural infection, as it is not vaccine-induced, helping distinguish infection from vaccination. Neutralizing antibodies, also targeting the RBD, signify functional immunity by blocking viral entry, reflecting protection against infection. Together, these measurements assess exposure history, immunity sources, and population-level outbreak risk [17].

Recent studies in Ghana show high SARS-CoV-2 seroprevalence, ranging from 67% to 87% in urban areas like Accra, reflecting widespread exposure through infection or vaccination [18,19]. Urban settings consistently exhibit higher seroprevalence than rural areas, likely due to greater population density, mobility, and access to healthcare [18,20]. However, lower seroprevalence among older adults aged ≥60 years and rural populations indicates potential vulnerability to future outbreaks, highlighting the need for ongoing surveillance and targeted vaccination to sustain long-term immunity in both urban and rural settings [18]. Furthermore, antibody waning over time underscores the importance of ongoing surveillance and targeted vaccination strategies, particularly in rural and young adults (20–39 years), who represent a high-risk group due to their greater mobility, social mixing, and occupational exposure [18,19].

Understanding urban-rural differences in serological responses is essential for identifying disparities in exposure risk and immune protection. In Ghana, geographic variation across broad regions (including northern and southern settings) co-exists with settlement-type differences (urban versus rural communities) within regions. In this manuscript, ‘north–south’ refers to geographic location, whereas ‘urban–rural’ refers to community type [21]. This study explores serological responses to SARS-CoV-2 in urban and rural Ghana, with a focus on antibody waning and its implications for sustained surveillance and public health interventions.

## 2. Materials and methods

### 2.1. Study design

The study utilised a household-based cross-sectional and longitudinal design to assess *SARS*-CoV-2 serological responses, serum samples collected at Time-point-1 (1^*st*^ August 2023–2^*nd*^ February,2024) with follow-up serum sampling at Time-point-2 (10^*th*^ August 2024–20^*th*^ February,2025) after one year. The research was carried out across five purposively selected communities in Ghana: Kumasi and Anyimadukrom near Obuasi (Ashanti Region), Buoyem and Forikrom (Bono East Region), and Tamale (Northern Region). These sites were chosen based on Ghana Health Service classifications as *COVID*-19 hotspots (Kumasi, Anyimadukrom) and coldspots (Tamale, Buoyem, Forikrom), based on district-level case burdens, which identified the Ashanti region as a major transmission hotspot during the national outbreak response. These represent a mix of urban (Kumasi, Tamale) and rural (Anyimadukrom, Buoyem, Forikrom) settings with varying population densities and socioeconomic characteristics. Community entry was facilitated through engagements with local health directorates, chiefs, and opinion leaders to ensure community acceptance and participation.

**Fig 1:**
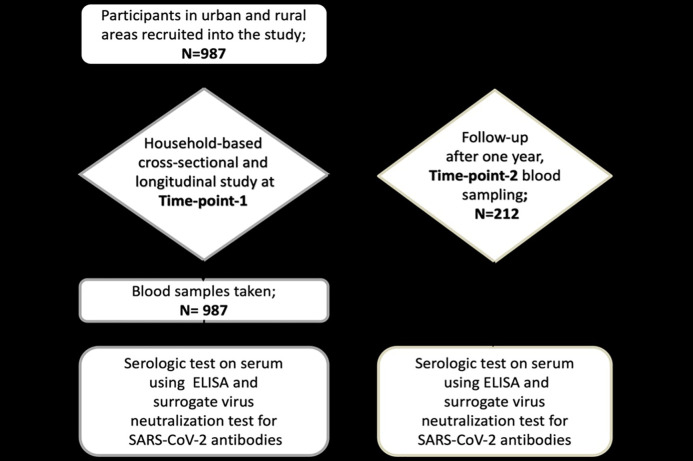
Flow chart of the overall study design and sampling workflow. The diagram summarizes participant recruitment and blood sampling at two time-points. At Time-point 1, 987 individuals from urban and rural households were enrolled in a cross-sectional and longitudinal study, and serum samples were collected for ELISA and surrogate virus neutralization testing. After one year, 212 participants returned for Time-point 2 follow-up sampling, and the same serologic assays were performed to assess SARS-CoV-2 antibody responses over time.

The study employed a two-phase approach (Fig 1). Initially, a cross-sectional survey at Time-point-1 involved random household sampling of community residents aged ≥10 years, regardless of respiratory symptoms, to establish baseline seroprevalence. Eligible participants were those residing in the selected households/communities who provided written informed consent (and assent where applicable) and agreed to venous blood sampling and questionnaire completion; individuals who declined consent/assent or blood sampling were not enrolled. The longitudinal phase followed consenting participants after one year (Time-point-2). Sampling was conducted by teams from the Kumasi Centre for Collaborative Research in Tropical Medicine (*KCCR*), using trained medical staff to collect venous blood samples (3–5 mL). Samples were processed and stored at -80^*o*^C following strict cold chain protocols for laboratory analysis at *KCCR*. Participants testing positive for *SARS*-CoV-2 at baseline were revisited after a one-year period to monitor seroconversion status, employing randomization within households to ensure representativeness across the selected communities. The Committee on Human Research, Publication and Ethics at Kwame Nkrumah University of Science and Technology authorised approval for the study with the following reference numbers: *CHRPE/AP*/292/23, *CHRPE/AP*/254/24, *CHRPE/AP*/401/25.

### 2.2. Sample size determination

The cross-sectional study sought to determine the difference in seroprevalence between rural and urban areas after time-point-1 sampling. To calculate the appropriate sample size to determine the difference in seroprevalence for the two populations, the method described by Wang & Chow, 2007 [22], was used with a confidence level of 95% and a power of 80%. This was based on statistical power calculations to identify significant variations with adequate precision.The expected seroprevalences for the rural and urban areas were deduced from prior studies done in Ghana [23–25]. The sample size was calculated to detect a prespecified difference in seroprevalence between comparison groups, assuming an estimated baseline seroprevalence, a two-sided significance level of 0.05, and statistical power of 80%. Detailed derivation of these assumptions is provided in the Supplementary Material.

### 2.3. Data capture and management

Data were collected by trained fieldworkers using structured questionnaires administered through REDCap (Research Electronic Data Capture), a secure web-based platform designed for clinical and research data management [26] The questionnaires captured demographic details (e.g., age, sex, and location), clinical data (e.g., respiratory symptoms), and vaccination information verified by vaccination cards (e.g., status, and dates). Fieldworkers underwent comprehensive training to ensure data accuracy, supported by regular quality checks. Written informed consent or assent was obtained from all participants prior to data collection and sample acquisition. Data from laboratory testing were also recorded in REDCap and all data were cleaned using Microsoft Excel.

### 2.4. Statistical investigations

Descriptive statistics was used to analysed the data, reporting categorical variables as frequencies and percentages, and continuous variables as medians. Associations between socio-demographic factors and SARS-CoV-2 seropositivity were assessed using Pearson’s Chi-square or Fisher’s exact test, with a significance level of p < 0.05, and 95% confidence intervals (CIs). Then, McNemar’s test, a paired statistical test used to evaluate whether there is a significant change in a binary outcome between two related time points, was performed to assess seroconversion status by comparing participants who will still maintain their dual S&N positivity at baseline and after one year follow-up. This analysis was conducted using STATA version 16.

### 2.5. Quantification of SARS-CoV-2 Anti-S IgG and Anti-N antibodies

Seropositivity was defined according to the manufacturer’s validated assay cutoff thresholds, whereby optical density index values below the lower cutoff were classified as seronegative, values within the indeterminate range as borderline, and values above the upper cutoff as seropositive, in accordance with kit instructions and quality-control procedures.SARS-CoV-2 Anti-S and -N IgG antibodies were measured using a commercial ELISA kit (Euroimmun GmbH, Lübeck, Germany). All serum samples were heat-inactivated at 56 °C for 30 min, diluted, and processed following the manufacturer’s instructions as previously reported [27]. The diluted samples, calibrators, and controls were added to antigen-coated microplate wells and incubated to allow antibody binding, followed by washing and incubation with a peroxidase-labelled anti-human IgG conjugate. After a subsequent wash, TMB substrate was added, and the reaction was stopped with sulfuric acid. Optical density (OD) was measured at 450 nm with a reference wavelength between 620–650 nm using a TEACAN microplate reader with Magellan software. Results were interpreted semi-quantitatively by calculating the ratio of sample OD to calibrator OD.

### 2.6. Detection of SARS-CoV-2 neutralizing antibodies

Neutralizing antibodies were detected using the cPass SARS-CoV-2 Surrogate Virus Neutralisation Test (sVNT) kit (GenScript Biotech, NJ, USA) to assess functional immunity rather than solely confirming IgG positivity. This assay identifies antibodies that block the SARS-CoV-2 spike protein's receptor-binding domain (RBD) interaction with the human ACE2 receptor, independent of species or isotype. A total of 148 participants were randomly selected at TP1 for neutralizing antibody testing, and a subset of 12 of these participants was followed up after one year to assess changes in percent neutralization. Serum samples were diluted 1:9 with the sample dilution buffer, mixed 1:1 with the HRP-conjugated RBD, and then incubated at 37°C for 30 minutes to allow antibody binding. Subsequently, 100 µL of each mixture was added to ACE2-precoated wells and incubated at 37°C for 15 minutes. Unbound material was removed with four 260 µL washes, followed by addition of 100 µL TMB substrate under reduced light at room temperature. The reaction was stopped with 50 µL stop solution, and absorbance was measured at 450 nm. Samples with ≥30% inhibition were deemed positive for neutralizing antibodies.

## 3. Results

### 3.1. Sociodemographic and clinical characteristics of study participants

The calculated minimum sample size to detect differences in SARS-CoV-2 seroprevalence between urban and rural areas was 60 participants per site. To enhance the study’s statistical power and account for potential dropouts or non-response, a total of 987 serum samples were collected at Time-point-1 (baseline), distributed as urban (640, 64.8%) and rural (347, 35.2%) areas. Of the 987 participants enrolled at baseline, 212 completed follow-up sampling. Non-participation at follow-up reflected participant unavailability or refusal of follow-up sampling; specific motivations for non-participation were not systematically collected.

Table 1 presents the sociodemographic characteristics of the 987 participants at Time-point 1. Age distribution showed a predominance of younger adults (20−29 years, 26.6%), while older age groups (70–89 years) were less represented (4.8%). Females comprised 67.3% of the cohort, and most participants were married or cohabiting (54.9%). Education levels varied, with the largest group having primary education (45.4%), and occupation was predominantly informal (72.6%). These data provide a baseline to explore potential associations with SARS-CoV-2 seropositivity, and guide targeted public health interventions.

**Table 1:** Distribution of Sociodemographic Characteristics Among Study Participants.

Characteristic	Frequency (N = 987)	Percentage (%)
**Residence**		
Rural	347	35.2
Urban	640	64.8
**Age group (years)**		
10-19	86	8.7
20-29	263	26.6
30-39	204	20.7
40-49	176	17.8
50-59	139	14.1
60-69	72	7.3
70-79	43	4.4
80-89	4	0.4
**Gender**		
Male	323	32.7
Female	664	67.3
**Marital status**		
Single/Living alone	445	45.1
Married or cohabiting	542	54.9
**Education**		
No formal education	231	23.4
Primary	448	45.4
Secondary	262	26.6
Tertiary	46	4.6
**Occupation**		
Formal	37	3.8
Informal	717	72.6
Retired	22	2.2
Unemployed	211	21.4

*Proportions were determined with descriptive column statistics.

**Fig 2:**
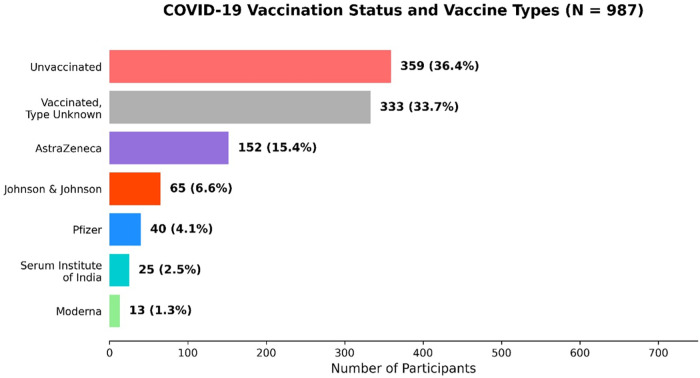
Distribution of COVID-19 vaccination status among study participants. Bar chart showing the distribution of vaccinated and unvaccinated individuals and the relative proportions of specific vaccine products. Vaccinated, type unknown represents participants who reported vaccination, but their vaccine cards were missing and could not confirm the specific brand. Values shown are counts with corresponding percentages.

Fig 2 summarise the self-reported vaccination status of the cohort (*N* = 987) and confirmed from their COVID-19 vaccination cards issued by the Ghana Health Service. Overall, 63.6% (*n* = 628) were vaccinated and 36.4% (*n* = 359) were unvaccinated. Among vaccinated participants confirmed by their vaccination cards, the most frequently administered product was reported products were AstraZeneca (15.4%, *n* = 152), Johnson & Johnson (6.6%, *n* = 65), Pfizer (4.1%, *n* = 40), Serum institute of India (2.5%, n = 25) and Moderna (1.3%, *n* = 13); for 33.7% (*n* = 333) the vaccine type was unknown.

### 3.2. Seroprevalence of SARS-CoV-2 at different timepoints among urban and rural populations in Ghana

**Fig 3:**
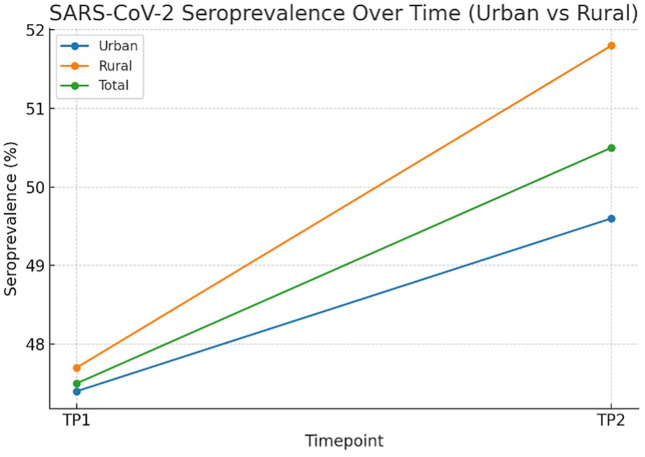
SARS-CoV-2 seroprevalence over time in urban and rural communities. This figure presents a line plot illustrating the proportion of participants with dual anti-spike (S) and anti-nucleocapsid **(N)** IgG seropositivity at baseline (TP1) and follow-up (TP2) in urban and rural areas. Points represent group-level point prevalence estimates (%), with lines connecting the timepoints, but 95% confidence intervals (CIs), calculated using the normal approximation method are provided for clarity.

Fig 3 illustrates the temporal evolution of SARS-CoV-2 seroprevalence in urban and rural communities. For urban participants, seroprevalence was 47.4% (95% CI: 43.5%–51.3%; n = 637) at time point 1 and 49.6% (95% CI: 40.9%–58.3%; n = 128) at TP2. For rural participants, seroprevalence was 47.7% (95% CI: 42.4%–53.0%; n = 346) at TP1 and 51.8% (95% CI: 41.2%–62.4%; n = 85) at TP2. Overall, seroprevalence was 47.5% (95% CI: 44.4%–50.6%; n = 983) at TP1 and 50.9% (95% CI: 43.8%–57.2%; n = 212) at TP2. Changes between TP1 and TP2 were modest (Δ: urban +2.2 percentage points; rural +4.1; total +3.4) and not statistically significant by χ² tests (urban: χ² = 0.13, p = 0.718; rural: χ² = 0.301, p = 0.58; total: χ² = 0.504, p = 0.478). TP1, baseline; TP2, follow-up; S, spike; N, nucleocapsid.

### 3.3. Association between participant characteristics and SARS-CoV-2 seroprevalence

Statistical analysis revealed a significant association between gender and seropositivity to the SARS-CoV-2 spike protein (Table 2). Specifically, females demonstrated a significantly higher seropositivity rate [648 (99%)] compared to males [306 (97%); p = 0.017]. In contrast, no significant associations were observed between any other participant characteristics and seropositivity to the nucleocapsid protein. Although other variables did not reach statistical significance, there was an observed trend suggesting that older individuals and those with diabetes may have a higher likelihood of being seropositive for SARS-CoV-2.

**Table 2:** Sociodemographic Characteristics and SARS-CoV-2 S and N Protein Antibody Status.

Characteristics	Borderline n/N (%)	S Negative n/N(%)	Positive n/N (%)	p-value^1^	Borderline n/N (%)	N Negative n/N(%)	Positive n/N(%)	p-value^2^
**Residence**				>0.9				0.8
Rural	2/344 (0.6)	3/344 (0.9)	339/344(99)		38/343(11)	141/343 (41)	164/343 (48)	
Urban	4/625 (0.6)	6/625 (1.0)	615/625(98)		61/627 (9.7)	268/627 (43)	298/627 (48)	
**Gender**				0.017				0.2
Female	2/653 (0.3)	3/653 (0.5)	648/653(99)		63/654 (9.6)	289/654 (44)	302/654 (46)	
Male	4/316 (1.3)	6/316 (1.9)	306/316(97)		36/316 (11)	120/316 (38)	160/316 (51)	
**Age group**				0.9				
10–19 yrs	1/234 (0.4)	1/234 (0.4)	232/234(99)		23/233 (9.9)	92/233 (39)	118/233 (51)	
20–29 yrs	2/223 (0.9)	2/223 (0.9)	219/223(98)		26/223 (12)	102/223 (46)	95/223 (43)	
30–39 yrs	1/187 (0.5)	2/187 (1.1)	184/187(98)		17/189 (9.0)	74/189 (39)	98/189 (52)	
40–49 yrs	1/137 (0.7)	2/137 (1.5)	134/137(98)		17/137 (12)	58/137 (42)	62/137 (45)	
50–59 yrs	0 (0)	2/92 (2.2)	90/92 (98)		10/92 (11)	37/92 (40)	45/92 (49)	
60–69 yrs	1/50 (2.0)	0 (0)	49/50 (98)		4/50 (8.0)	24/50 (48)	22/50(44)	
70–79 yrs	0 (0)	0 (0)	36/36 (100)		2/36 (5.6)	17/36 (47)	17/36 (47)	
80–89 yrs	0 (0)	0 (0)	10/10 (100)		0 (0)	5/10 (50)	5/10 (50)	

n: number of participants in each antibody category, N: total number of participants within the respective subgroup, n/N(%): proportion within subgroup, ^1^Fisher’s exact test was used for anti-spike(S) antibody comparisons due to small cell counts, ^2^Pearson’s Chi-square test was used for anti-Nucleocapsid (N) antibody comparisons, S: anti-Spike antibody results reflecting infection and/or vaccine-induced responses, N: anti-Nucleocapsid antibody results reflecting infection-induced immunity only. Denominators for subgroup percentages reflect participants with valid serological results for the corresponding antibody measurement. Individuals with missing or indeterminate serology were excluded from subgroup-specific analyses; therefore, subgroup totals may differ from overall demographic counts presented in Table 1.

### 3.4. Dual S and N antibody dynamics and neutralization assays over one year

Among the 212 participants (Table 3), who were dual anti-spike (S) and anti-nucleocapsid (N) IgG positive at baseline (Time-point-1) and consented to follow-up (Time-point-2), longitudinal analysis revealed substantial within-individual antibody waning over one year. While all participants were dual-positive at baseline by definition, only 50.9% (108/212; 95% CI: 44.0–57.4) remained dual-positive at follow-up, with 49.1% (104/212) reverting to seronegative status. This within-participant antibody decline was statistically significant (McNemar’s χ² = 104.0, df = 1, p < 0.0001), demonstrating substantial waning of naturally acquired antibodies over time despite stable population-level seroprevalence.

**Table 3:** Changes in Combined S and N Antibody Seroprevalence over Time in Urban and Rural Populations.

		Tested	Number positive			Seroprevalence	Proportions	
**Area**	TP	n	S	N	S&N	S&N (%)	TP2 - TP1 (%)	P-value (95% CI)
**Urban**	1	637	625	304	302	47.4%	2.2%	0.718 (−0.121-0.0772)
	2	128	128	64	64	49.6%		
**Rural**	1	346	341	166	165	47.7%	4.1%	0.58 (−0.1667-0.085)
	2	85	84	44	44	51.8%		
**Total**	1	983	966	470	467	47.5%	3.4%	0.478 (−0.106-0.047)
	2	212	212	108	**108**	50.9%		

S: Spike, N: Nucleocapsid, S&N: Dual Spike and Nucleocapsid, TP: Time point, CI: Confidence intervals, n: Sample, %: Percentage.

**Fig 4:**
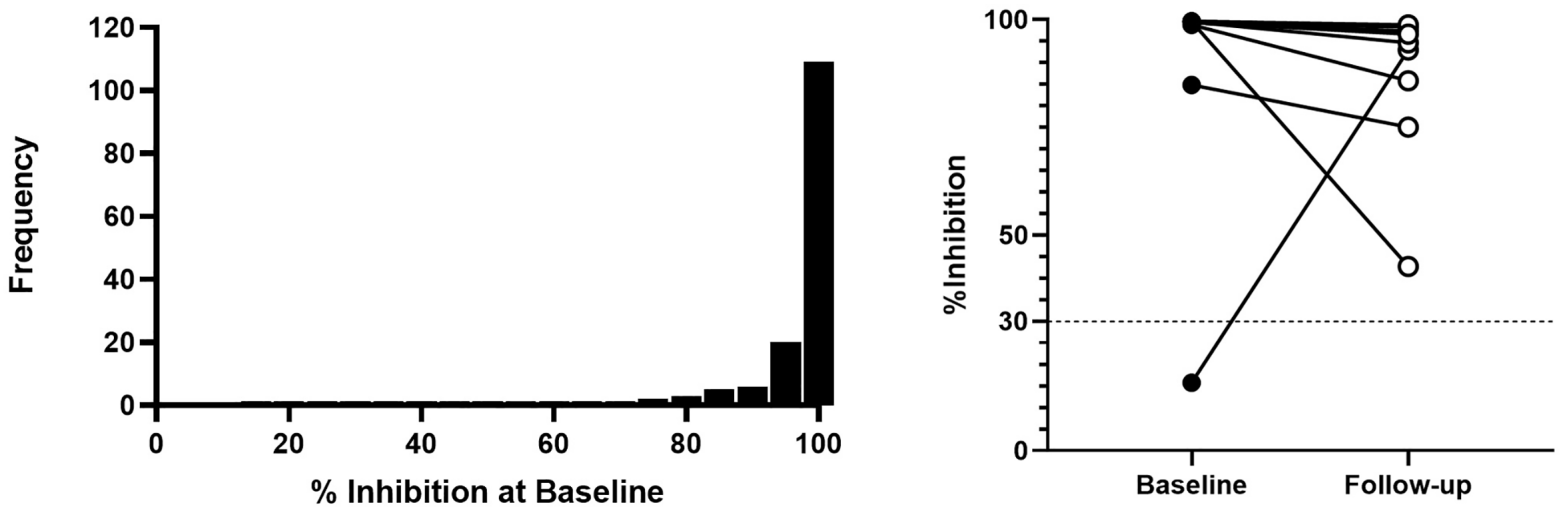
Baseline and longitudinal inhibition profiles of selected participants. Panel A, shows the distribution of percentage inhibition values at baseline, illustrating the variability in neutralising antibody activity across participants. Panel B presents paired baseline and one-year follow-up inhibition values, with connecting lines depicting individual-level changes and highlighting the overall decline in neutralising activity over time. The dashed horizontal line indicates the 30% inhibition threshold for neutralising antibody positivity.

Fig 4 illustrates the distribution and longitudinal changes in SARS-CoV-2 neutralising antibody activity. Fig 4A (Panel A), shows the distribution of percentage inhibition values at baseline (Time-point-1), indicating substantial inter-individual variability in neutralising antibody responses. Fig 4B (Panel B), presents paired baseline and one-year follow-up (Time-point-2) inhibition values for the same selected participants, with connecting lines indicating within-participant changes over time. Most participants demonstrated a clear decline in inhibition levels over the one year, consistent with waning neutralising antibody activity. However one participant had inhibition below the 30% inhibition positive cut-off point at baseline but changed to positive inhibition at follow-up, which may be due to infection or vaccination.

### 3.5. Immune response markers stratified by vaccination status

**Fig 5:**
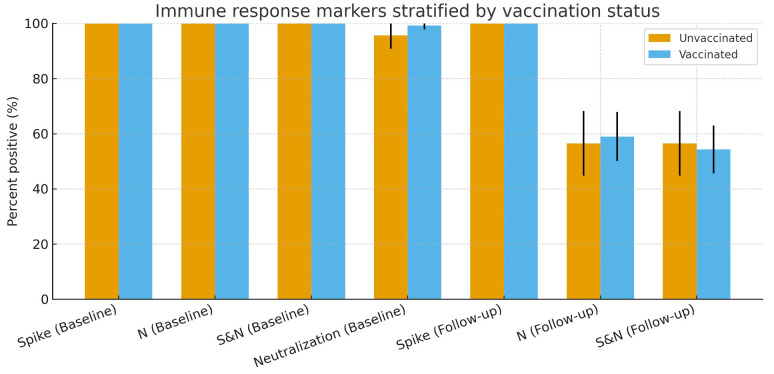
Immune response markers by vaccination status. Bars show the proportion positive (%) for spike (S), nucleocapsid (N), dual S&N, and neutralization at baseline and follow-up, stratified by vaccination; error bars denote 95% CIs. At baseline, neutralization was higher in vaccinated (99.3%) than unvaccinated (95.7%). At follow-up, S remained 100% in both groups, while N was 59.0% (95% CI 50.1–67.9) vs 56.5% (44.8–68.2), and S&N was 54.3% (45.7–63.0) vs 56.5% (44.8–68.2) in vaccinated vs unvaccinated, respectively, consistent with persistence partly attributable to vaccine-induced immunity.

Only 29.9% (295/987) of participants had vaccination status confirmed by vaccination cards, yet immune response markers showed clear differences between vaccinated and unvaccinated groups as seen in Fig 5. At baseline, neutralisation positivity was slightly higher in vaccinated individuals (99.3%) compared with the unvaccinated (95.7%), despite similarly high spike, nucleocapsid, and dual S&N positivity in both groups. At follow-up, spike IgG remained 100% across groups, whereas N and dual S&N positivity declined similarly in vaccinated (59.0% and 54.3%) and unvaccinated (56.5% and 56.5%) participants. These patterns suggest that sustained spike responses may reflect vaccine-derived immunity; whereas the decline in N and dual S&N positivity over time is consistent with waning infection-induced antibodies.

## 4. Discussion

The study included participants from both urban (Tamale, Kumasi) and rural (Forikrom, Anyimadukrom, Buoyem) study sites in Ghana. This depicts a deliberate effort to capture geographic and demographic diversity, which is crucial for understanding the spread and impact of SARS-CoV-2. Empirically, SARS-CoV-2 seroprevalence at baseline was comparable between urban (47.4%) and rural (47.7%) communities, indicating widespread exposure across settings during the early post-pandemic phase. At follow-up, seroprevalence increased modestly in both areas to 49.0% in urban and 51.8% in rural populations, suggesting continued community transmission and possible vaccine-induced antibody boosting. The differences were small, the slight rise in rural seropositivity may be difficult to distinguish between infected and vaccinated due to loss of anti-N antibodies [28]. The N antibodies arise exclusively from natural infections and wane more rapidly, whereas the S antibodies persist longer following infection or vaccination and may be boosted by repeated exposure. Similar approaches in large-scale seroprevalence studies have shown that urban-rural differences, population density, and local COVID-19 incidence can significantly influence exposure risk and antibody prevalence [29–31]. The use of ELISA diagnostic methods strengthens the reliability of past infection and immunity assessments, as recommended in global seroprevalence protocols [29].

Recent population-based studies in Ghana have demonstrated high SARS-CoV-2 seroprevalence, with national estimates ranging from 39.6% in July 2021 to 67.1% by the end of 2021, reflecting widespread exposure across the country [18,32]. In Greater Accra, seroprevalence reached as high as 86.8% during the Omicron wave, indicating intense community transmission and/or high vaccine-induced immunity [19]. In this study, seroprevalence increased modestly from 47.5% at the first time point to 50.9% one year later, with no statistically significant decline in antibody levels. This finding is consistent with other Ghanaian data showing sustained antibody responses over time, suggesting limited waning of humoral immunity within a one-year period [19,32]. Importantly, no significant difference in seroprevalence was observed between urban and rural participants. While some national surveys have reported higher seroprevalence in urban areas, attributed to greater population density and increased exposure risk, a similar pattern was observed in Ghana, where a nationally representative survey concluded that exposure is more likely in urban than rural settings [18]. Other studies, including this study, found that by late 2021, urban-rural differences had diminished, likely due to widespread community transmission and the impact of vaccination campaigns [18,32,33]. This convergence in seroprevalence may indicate that the virus had circulated extensively in both settings by the time of sampling, contributing to a form of population-level immunity. A higher proportion of participants had antibodies against the spike protein compared to the nucleocapsid protein, suggesting that vaccination, rather than natural infection, may have been the primary driver of seropositivity in the study population. This pattern aligns with findings from Accra, where vaccination status was strongly associated with seropositivity [19]. The overall vaccination coverage within the study population was 63.6% (628/987) as against 43.7% of Ghanaians who had received at least one dose of the vaccine by April,2024, which was within the study period [34]

The observed significant association between genders and higher seropositivity to the SARS-CoV-2 spike (S) protein (99% in females vs. 97% in males, p = 0.017) is notable and that represents the overall seroprevalence based on anti-S antibodies. Although statistically significant (p = 0.017), the small difference in seropositivity between females (99%) and males (97%) likely reflects the large sample size rather than a meaningful biological or clinical difference, indicating that statistical significance does not necessarily imply clinical relevance. S-antibodies are the most informative marker of population-level seroprevalence in the post-pandemic period, which will aid vaccination strategies targeted at vulnerable groups rather than broad, population-wide measures. However, large-scale meta-analyses and systematic reviews generally report no consistent difference in seroprevalence between genders [14,35]. For example, a global review found no statistically significant difference in seroprevalence between male and female subjects [35], and a meta-analysis of over 2.3 million participants similarly found no sex-based difference [14]. No significant associations were found between other participant characteristics (such as age or comorbidities) and seropositivity to the nucleocapsid protein. This aligns with several international studies, which often report that age and comorbidities are not consistently associated with seropositivity, especially after widespread vaccination [35,36]. However, some studies have observed trends toward higher seroprevalence in older adults and those with certain health conditions, though these trends frequently do not reach statistical significance [36,37]. For example, a Swiss population-based study found that after the vaccination campaign, differences in seropositivity by age and comorbidity disappeared when adjusting for vaccination status [36]. The trend toward higher seropositivity in older individuals and those with diabetes, though not statistically significant, is consistent with findings that these groups may have higher exposure risk or vaccine uptake [36,37]. Globally, other factors such as ethnicity, socioeconomic status, and occupational exposure have been more consistently associated with seroprevalence differences than gender or age alone [14,38,39]. The uniformity in seroprevalence observed may reflect widespread transmission of the virus by the time of sampling, which occurred post-pandemic, potentially contributing to a form of herd immunity. Notably, a higher proportion of individuals had detectable antibodies against the S protein compared to the N protein, implying that immunity in most participants may be more likely due to vaccination rather than natural infection.

The neutralising antibody activity was observed among participants with available follow-up samples, consistent with persistence of humoral immune responses after infection or vaccination [40]. However, a small number of individuals were randomly selected and had paired neutralisation measurements at the second time point (n = 12), and the longitudinal cohort overall represented a reduced subset of the baseline population (n = 212 of 987). These constraints substantially limit inference regarding the durability and distribution of functional immunity at the population level. Accordingly, the neutralisation findings should be interpreted as descriptive evidence within a limited exploratory subgroup rather than definitive evidence of sustained population-level protection. Larger longitudinal studies incorporating repeated functional immune measurements are required to robustly characterise long-term immunity in these settings. Similar declines in neutralising activity have been reported in longitudinal studies, which show that SARS-CoV-2 neutralising antibodies peak within weeks of infection and subsequently decrease, particularly in the absence of boosting by vaccination or reinfection [40,41]. The decline observed in our study therefore aligns with global evidence demonstrating that natural infection alone may not provide durable neutralising immunity, underscoring the importance of continued vaccination efforts to sustain protective antibody responses.

### 4.1. Limitations

The relatively small sample size for longitudinal and neutralisation analyses, potential attrition bias influencing follow-up interpretations, and the absence of cellular immune response assessment, limit a comprehensive evaluation of long-term immunity.

## 5. Conclusion

This study demonstrates widespread serological evidence of prior SARS-CoV-2 exposure and vaccine-induced immune responses across both urban and rural populations in Ghana. Functional neutralising activity was detectable within a limited longitudinal subset, suggesting persistence of antibody-mediated protection in some individuals. Nevertheless, the small number of participants with neutralisation follow-up measurements limits conclusions regarding the durability of functional immunity at the population level. Continuous longitudinal surveillance using larger cohorts and repeated functional immune assessment will be essential to clarify long-term protection in West African settings.

## Supporting information

S1 File Serology Data. (XLSX)

S2 File Sample size calculation determination. (DOCX)

S3 File Inclusivity in global research. (DOCX)
