# Supplementary material for: Serological responses to SARS-CoV-2 in urban and rural Ghana: Antibody waning and implications for long-term population immunity
Source: PLoS One. 2026 May 28;21(5):e0348281. doi: 10.1371/journal.pone.0348281 (PMC13218506; doi:10.1371/journal.pone.0348281)
Supplement: S2 File — (DOCX) [file pone.0348281.s002.docx]

**SAMPLE SIZE CALCULATION DETERMINATION**

The study aimed to compare SARS-CoV-2 seroprevalence between urban and rural populations in Ghana at baseline (time-point 1) and at follow-up, focusing on participants with dual antibody positivity (anti-spike and anti-nucleocapsid) at time-point 2, one year later. The baseline assessment aimed to identify differences in seroprevalence between rural (p1) and urban (p2) areas following initial sampling, to evaluate whether COVID-19 mitigation measures and vaccination efforts were equally implemented across these regions. The longitudinal component involved re-sampling SARS-CoV-2 positive participants at time-point 2 to determine their seroconversion status after one year.

To calculate the appropriate sample size to determine the difference in seroprevalence for

the two populations, the method described by (Wang & Chow,2007), was used; $n={(z_{\alpha/2}+z_{\beta})}^{2}\times\frac{(p_{1}(1-p_{1}) +p_{2}(1-p_{2}))}{{(p_{1}-p_{2})}^{2}},$where α is type-1 error, β is type-2 error, Z_α/2_ is the critical value of the normal distribution at α/2 (confidence level of 95%, α is 0.05 and the critical value is 1.96), Z_β_ is the critical value of the normal distribution at β (for a power of 80%, β is 0.2 and the critical value is 0.84) and p_1_ and p_2_ are the expected seroprevalence of the two groups that is rural and urban $n=\left( 1.96+0.84 \right)^{2}*\frac{(0.186(1-0.186)+0.412(1-0.412)}{{( 0.186-0.412)}^{2}}=60.4, n= 60.$

The expected seroprevalence for the rural (p_1_) was deduced after looking at this study done in Ghana which is, 18.6% in Akropong (Quashie et al.,2021). The 18.6% was assumed an expected rural seroprevalence, using Akropong (population: 15,274; Akuapem North, Eastern Region) and Anyimadukrom in Obuasi East (rural population: 22,960) as representative rural communities.

(Ghana Statistical Service, 2021).

The seroprevalence for the urban area (p_2_) was reported as 41.2% in Kumasi (Struck et al., 2022). This value was used as the expected seroprevalence because Kumasi is one of the urban sites in the study. To improve sampling efficiency, the finite population correction (FPC) formula (Lavrakas, 2013) was used to determine the minimum sample sizes needed from both urban and rural areas, considering that the total populations of the three rural areas, Obuasi-east (22,960), Forikrom (4,470), and Buoyem (4,587)—are below 100,000. In contrast, the urban populations in Kumasi (443,981) and Tamale (374,744) exceed 100,000.

To apply a finite population correction to the sample size calculation for comparing the difference in the seroprevalence for the two groups above, we will include f_1_=(N_1_-n)/(N_1_-1) and f_2_=(N_2_-n)/(N_2_-1) in the formula as follows, substituting f_1_ and f_2_ into the formula below, we get the following,

$n={(z_{\alpha/2}+z_{\beta})}^{2}\times\frac{(p_{1}(1-p_{1}) +p_{2}(1-p_{2}))}{{(p_{1}-p_{2})}^{2}}$

$n={(z_{\alpha/2}+z_{\beta})}^{2}\times\frac{(f_{1 \times}p_{1}(1-p_{1}) +{f_{2 \times}p}_{2}(1-p_{2}))}{{(p_{1}-p_{2})}^{2}} n=X \times\frac{A}{(1+X\times B)}$ , where;

$X=\frac{{(z_{\alpha/2}+z_{\beta})}^{2}}{{(p_{1}-p_{2})}^{2}}$ ,

$A=({N_{1}}/{(N_{1}-1}))\times{(p}_{1}(1-p_{1}))+({N_{2}}/{(N_{2}-1}))\times{(p}_{2}(1-p_{2})),$and

$B=(1/{(N_{1}-1}))\times{(p}_{1}(1-p_{1}))+(1/{(N_{2}-1}))\times{(p}_{2}(1-p_{2})),$and;

Z_α/2_ = 1.96, Z_β_ = 0.84, p_1_ = 18.6% = 0.186, p_2_ = 41.2% =0.412

N_1_(Rural areas, Anyimadukrom, Forikrom and Buoyem: 22,960+4470+4587) = 32,017. N_2_(Urban areas, Kumasi & Tamale: 443,981+374,744) = 818,725.

Using the above values, the estimates for X, A, and B will be:

X= 153.5, A= 0.394, B = 5.02 x 10^-6^

Then, the minimum sample size (n) can be calculated

n = X * A / (1 +X * B), n =153. * 0.394 / [1 + (153.5 * 5.02 * 10^-6^] = 60.4, n = 60.

Therefore, taking a minimum of 60 samples from each of the study site(s) will be enough to determine the difference in seroprevalence for the rural and urban areas.
